# Detection of Cu^2+^ in Water Based on Histidine-Gold Labeled Multiwalled Carbon Nanotube Electrochemical Sensor

**DOI:** 10.1155/2017/1727126

**Published:** 2017-03-20

**Authors:** Rilong Zhu, Gangqiang Zhou, Fengxia Tang, Chunyi Tong, Yeyao Wang, Jinsheng Wang

**Affiliations:** ^1^College of Water Sciences, Beijing Normal University, Engineering Research Center for Groundwater Pollution Control and Remediation, Ministry of Education, Beijing 100875, China; ^2^Hunan Province Environmental Monitoring Centre, Protection Key Laboratory of Monitoring for Heavy Metal Pollutants State Environmental, Changsha 410014, China; ^3^College of Biology, Hunan University, Changsha 410082, China; ^4^China Environmental Monitoring Centre, Beijing 100012, China

## Abstract

Based on the strong interaction between histidine and copper ions and the signal enhancement effect of gold-labeling carbon nanotubes, an electrochemical sensor is established and used to measure copper ions in river water. In this study the results show that the concentrations of copper ion have well linear relationship with the peak current in the range of 10^−11^–10^−7 ^mol/L, and the limit of detection is 10^−12 ^mol/L. When using this method to detect copper ions in the Xiangjiang River, the test results are consistent with the atomic absorption method. This study shows that the sensor is convenient to be used in daily monitoring of copper ions in river water.

## 1. Introduction

Copper is most important metal widely used in chemical industries, mining industry, and so on and ranked the third most abundant metal ion (after Fe^2+^ and Zn^2+^) among the essential heavy metal ions in mammals and acts a vital role in different processes of fundamental physiology in organisms, varying from bacteria to mammals [1]. However, excess or insufficient copper can cause many diseases, such as Wilson's disease, Alzheimer's disease, and Menke's disease [2]. Due to the important roles of copper ions in industrial, environmental, and biological processes, it is on the main daily monitoring list of civil water all the time. So, there is a great demand for the development of simple, selective, and sensitive methods for copper ions [3]. Currently there are different methods that have been developed for the detection of copper ions [4–7]. Most widely used methods for detection of copper are traditional atomic spectrometry, UV-Vis spectrometry, and electrochemical analysis. Among these methods, electrochemical analysis has received the widespread attention due to its simpleness, low cost, easy to carry, and so on. Some studies have shown that through different chemical modification of the electrodes, the specific sensitive detection for different heavy metals can be successfully implemented [8, 9]. With the development of nanomaterials, developing a suitable electrode modification layer is helpful and necessary.

Multiwalled carbon nanotubes (MWCNT) have received great attention because of their very large specific surface area, porous structure, and good electrical conductivity [10]; It is often used for modification onto the electrode to strengthen the electrode performance and is used as basement to cross-link other compounds to the electrode. Zhao et al. [11] achieved electrical aggregation of 2-amino-4-thiazoleacetic acid onto multiwalled carbon nanotubes, thereby realizing the detection of the copper ions, and the detection limit is 5.0 × 10^−10 ^mol/L; Saidin et al. [12] report on a MWCNT paste electrode modified with the fenchone diazine tetracarbonylmolybdenum(0) complex, to sensitive determination of Cu(II) by using square wave anodic stripping voltammetry; the limit of detection is 80 pM; Fu et al. [13] modified single-walled carbon nanotube on the electrode, and on the basis of carbon nanotubes, electrodepositing a layer of gold nanoparticles, and then according to Au-S bonding modifying L-cysteine onto the electrode, so as to realize the detection of heavy metal ions, the limit of detection was found to 0.02 nM. The reports show that the nanomaterials modified multiwalled carbon nanotube can be used for detecting metal ions, and the limit of detection is lower than that without modification.

In addition to increasing the specificity, some biomolecules such as histidine and BSA are used to modify on the carbon nanotubes. Because of having strong capacity to coordinate with copper ion, histidine is usually used for specific detection of copper. Yang et al. [14] combined tripeptide Gly-Gly-His on the gold electrode through Au-S binding, the peptide modified electrodes exhibited high sensitivity to copper, and when applied to detect copper ions, the limit of detection is 0.2 ppm. By the same idea, Li et al. [15] modified the gold electrode with mercaptoethylamine and histidine by using glutaraldehyde, realizing the specific detection of heavy metal copper and the detection limit is lower to 0.5 × 10^−12^ mol/L. So, the proper modification with histidine may increase the specificity of copper ions and enhance the detection sensitivity.

Therefore, based on the strong binding between histidine and copper ion and the good electrical conductivity of multiwalled carbon nanotubes, here we establish a complex modified electrode and use it to detect copper ions from Xiangjiang River water.

## 2. Experimental

### 2.1. Reagents and Instruments

L-Histidine (Shanghai Sangon Biological Engineering Co., Ltd); 1,6-ethanethiol (Sigma Company Inc.); HAuCl_4_·4H_2_O; MWCNT (Chengdu Organic Nanoparticles Company) reagents were used without further purification and water in experiments was ultrapure water which was prepared by Millipore ultrapure water system.

All electrochemical measurements were completed by CHI660D electrochemical workstation (Shanghai CH Instruments, Inc.). Use glassy carbon electrode (*d* = 3 mm, Shanghai CH Instruments, Inc.) as working electrode, platinum electrode as counter electrode, and saturated calomel electrode (SCE) as reference electrode to form a three-electrode system. Atomic absorption spectrometer AA6800 (Shimadzu Corporation) was used to test copper ion as reference method.

## 3. Procedures

### 3.1. Preparation of Gold-Labeling Multiwalled Carbon Nanotube

Gold-labeling multiwalled carbon nanotube (MWCNT/Au) was prepared as the method reported in the literature [16]. Firstly, gold chloride acid and trisodium citrate solution filtered through suction filtration with aperture 0.22 *μ*M; all glassware soaks were washed with aqua regia (*V*_HCl_ : *V*_HNO3_ = 3 : 1) and rinsed thoroughly by ultrapure water. Secondly, acid treated carbon nanotubes were suspended in 1% gold chloride acid solution to the last concentration of 1 mg/mL, dispersed 5 min by ultrasound, and then diluted to 100 mL with double deionized water; meanwhile they were mixed by magnetic stirring and heat till boiling. Thirdly, sodium citrate was added to the boiling solution; then the solution was heated 5–10 min until its color did not change. After all steps, the harvest solution can be stored at the temperature of 4°C after being cooled.

### 3.2. Modification of Glassy Carbon Electrode

The modification processes contained five steps: (a) glassy carbon electrode (GCE) pretreatment; (b) gold nanoparticle deposition on the surface of GCE; (c–e) the 1.6-ethanethiol, MWCNT/Au, and histidine layer-by-layer self-assembled on the surface of GCE. At the first step, the surface of GCE was polished by 0.3 and 0.05 *μ*m alumina powders, respectively, then washed in ultrapure water, ethanol, and ultrapure water for 1 min in turn to remove residual of alumina powders, and then scanned by cyclic voltammogram in 0.1 M acetic acid solution (pH = 6.0) within the voltage range of −0.8 to 0.8 V until getting a stable cyclic voltammetric peak and washed with ultrapure water. At the second step, the pretreatment electrodes were immersed in 0.04% HAuCl_4_ solution (dissolved in 0.01 M K_2_SO_4_) to electrochemical deposit for 60 s under the constant potential 0.2 V, and then the Au/GCEs were prepared. At the third step, the Au/GCEs were immersed into 1.6-ethanethiol solution (0.01 M, dissolved in anhydrous ethanol) for 16 hours under 4°C and then washed with ethanol and water in turn. At the fourth step, the pretreated electrodes were immersed in the prepared MWCNT/Au solution for 6 hours in room temperature and then washed out with ultrapure water. At the last step, the electrodes were immersed in 10 mM histidine solution for 6 hours in room temperature; then after washing three times by ultrapure water, the modified electrodes were prepared successfully. All steps were monitored by cyclic voltammograms and impedance.

### 3.3. Electrochemical Measurement

When they were used to measure copper ion, the composite electrodes were firstly immersed in the acetic acid solution (pH = 6.0) containing different concentrations of copper ion with magnetic stirring for three minutes. Then differential pulse stripping voltammetry was used to preenrich and reduct Cu(II) to Cu(0) on the surface of composite electrode. Subsequently, differential pulse voltammograms were record between −0.2 V and 0.4 V after static balanced 15 s. Some usable details were that the electrodes were immersed in 0.01 M of HNO_3_ solution for 60 s under 0.5 V to remove residual metal from the last experiment before each experiment and the washing time should be appropriately extending with the increasing of concentration to be measured.

## 4. Results and Discussion

### 4.1. Modification Process of Composite Modified Electrodes

**Figure 1:**
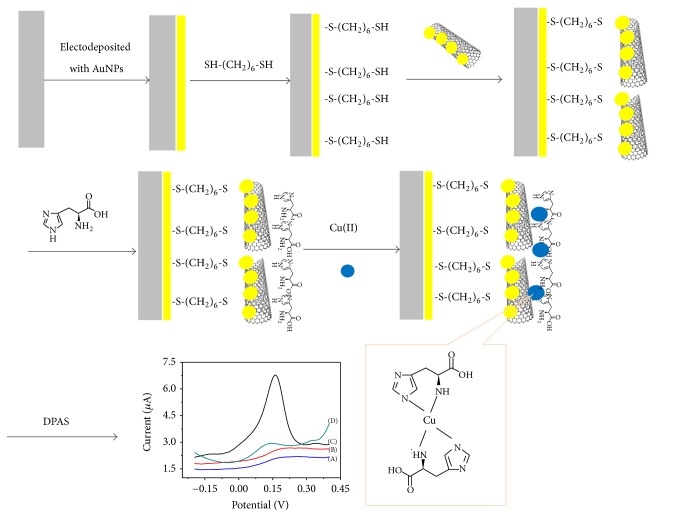
Schematic diagram of modification process of compound modified electrode.

The electrochemical sensor is based on the histidine's specific complexation with copper ion and the effect of enhanced signal of carbon nanotubes and gold nanoparticles. So, we modify these elements on the surface of glassy carbon electrode through the layer-by-layer self-assembly. The modification process is shown in Figure 1. First, deposit a layer of gold nanoparticles on the surface of glassy carbon electrode by electrodeposition. Then, use 1,6-ethanethiol to connect the gold nanoparticles modified electrode and gold-labeling multiwalled carbon nanotubes to make the carbon nanotubes on the surface of the electrode. Finally, histidine was absorbed on the electrode surface through electrostatic adsorption between amino on the histidine surface and gold nanoparticles. After layer-by-layer self-assembly modification, the compound modified electrode (CME) is completed and could be used for copper ions detection. Here, when the CME absorbed copper ions, the current will be changed, and the change is related to the enrichment of copper ions.

### 4.2. Characterization of Gold-Labeling Multiwalled Carbon Nanotubes

**Figure 2:**
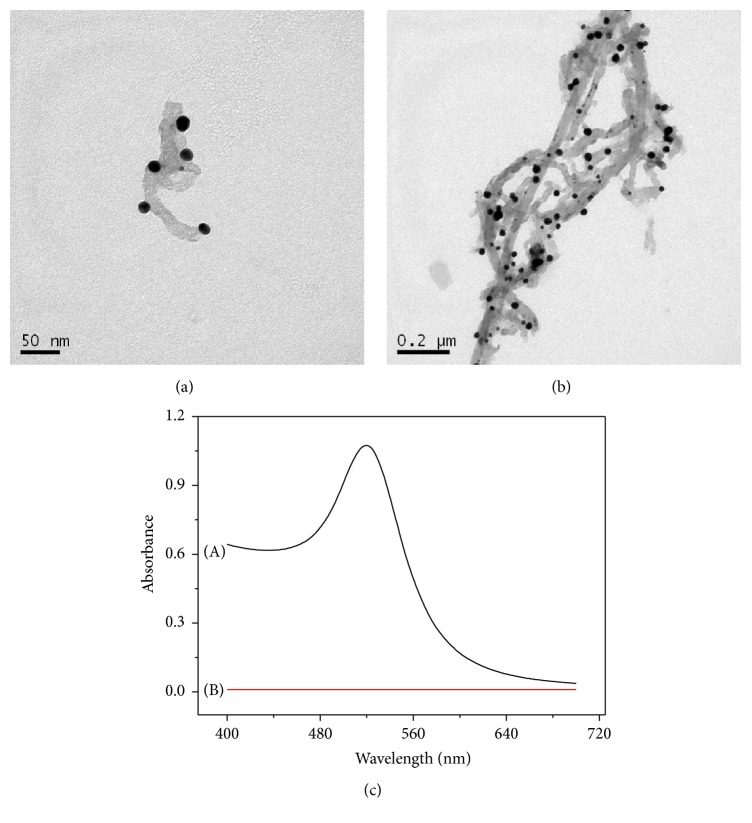
Characterization of MWCNT/AuNPs by SEM (a, b) and UV-Vis (c).

In order to modify the carbon nanotubes stably on the electrode surface by 1.6-ethanethiol, gold-labeling carbon nanotubes (MWCNT/AuNPs) were prepared. As shown in Figure 2, the scan electron microscopy (SEM) and ultraviolet spectrometry (UV-Vis) were used for characterization. From the SEM graph of Figures 2(a) and 2(b), gold nanoparticles (black dots) were marked successfully on the surface of carbon nanotubes. And the UV-Vis spectrum (Figure 2(c)) also showed that gold nanoparticles were labeled successfully. The pure carbon nanotubes had no obvious absorption peak within 400–700 nm (B), but an obvious absorption peak (A) at 522 nm appeared after gold-labeling.

### 4.3. Electrochemical Characterization of the Electrode Modification Process

**Figure 3:**
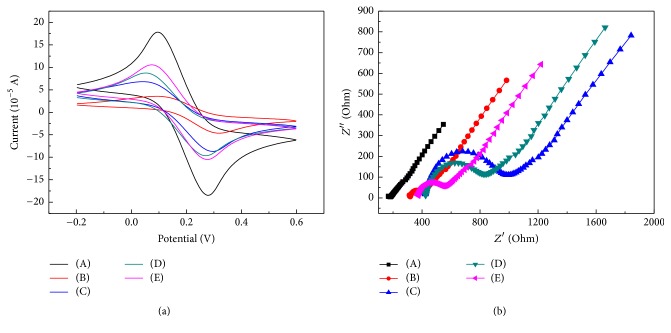
Electrochemical characterization of the modifying process by CV (a) and EIS (b) [resp., (A) bare GCE; (B) after deposited AuNPs; (C) after assembled SH; (D) after modified MWCNT/AuNPs; (E) after modified histidine].

Electrode layers upon layer self-assembly process were characterized by electrochemical method, using cyclic voltammograms and electrochemical impedance spectroscopy to characterize the process as shown in Figure 3. The modification processes were containing four steps, depositing gold nanoparticles, modifying 1.6-ethanethiol, modifying gold-labeling carbon nanotubes, and modifying histidine in turn. The cyclic voltammograms spectrum is shown in Figure 3(a), in which the whole processes presented regular cyclic voltammograms. With the modification processes, peak height and the properties of modified molecules displayed periodic variation. After depositing gold layers, gold nanoparticles with certain negative charge have electrostatic repulsion with the negatively charged [Fe(CN)_6_]^3−/4−^, thus blocking the electron transfer and reducing the peak certainly; after the self-assembly of 1,6-ethanethiol, the spike was further broaden and current intensity was further reduced; MWCNT/AuNPs had bigger specific surface area that made the electron transfer speed accelerated and the spike obvious; histidine carried with amino, protonated and positively charged, which benefit the transfer for the negatively charged [Fe(CN)_6_]^3−/4−^, thus getting good spike, providing places and conditions for further enrichment of copper ion. Moreover, electrochemical impedance spectrum of Figure 3(b) displayed the corresponding results of cyclic voltammograms. When electron transfer was blocked, impedance radius increased; however, when electron transfers smoothly, impedance radius decreased. After all, a series of electrochemical characterization showed that all chemicals were layer by layer self-assembled on the surface of electrode.

### 4.4. Feasibility for CME to Detect Copper Ions

**Figure 4:**
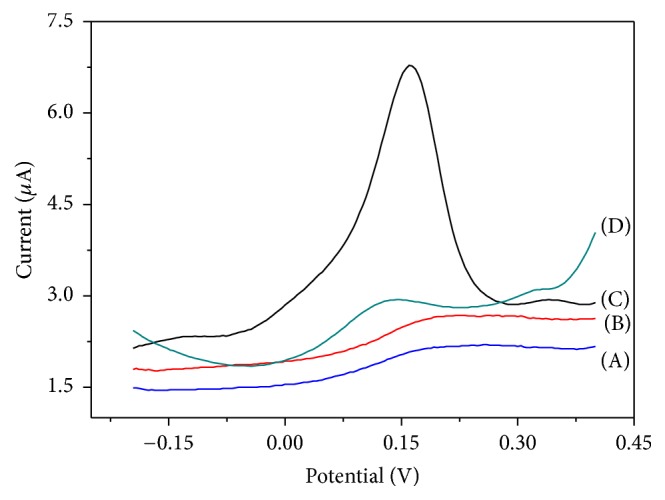
DPASV of detection of copper ions based on modified electrodes (C_Cu2+_ = 10^−8 ^mol/L). [(A) bare GCE; (B) AuNPs/MWCNT-SH/AuNPs/GCE; (C) histidine/AuNPs/MWCNT-SH/AuNPs/GCE; and (D) histidine/AuNPs/GCE].

To investigate that the whole modification is necessary, we compared the signal intensity of different modification levels. As shown in Figure 4, bare GCE, AuNPs/MWCNT-SH/AuNPs/GCE, histidine/AuNPs/GCE, and histidine/AuNPs/MWCNT-SH/AuNPs/GCE were compared by DPASV. The dissolution current intensity of copper ions on the electrode surface was different by different electrodes. Without histidine (lines (A) and (B)), there was no obvious peak, although the gold nanoparticles could enhance the current signal on the electrode. After modified histidine (line (C) and (D)), obvious current peaks emerged at 0.15 V. It means histidine absorbed copper ions on the surface of electrode. Meanwhile, after assembled AuNPs/MWCNT, the intensity increased significantly, about 2.5 times. That was because the AuNPs/MWCNT could increase the electron transport and its huge specific surface area and interspace provided places for the complexing of histidine and metal ion, and at the same time the carboxylic multiwalled carbon nanotubes could have complex reaction with metal ions, effectively improving the efficiency of the copper ion enrichment and thus resulting in sensitive stripping peak. In addition, compared with lines (C) and (D), although both electrodes are modified with AuNPs, the current intensity was different. The current signal of the electrode with MWCNT (C) was much higher than that without it (D). That means the MWCNT could enhance the current and made the electrode more sensitive. These data showed that CMEs could be used for the detection of copper ion, and the detection sensitivity was higher than separate histidine modification.

### 4.5. Optimization of Test Conditions

**Figure 5:**
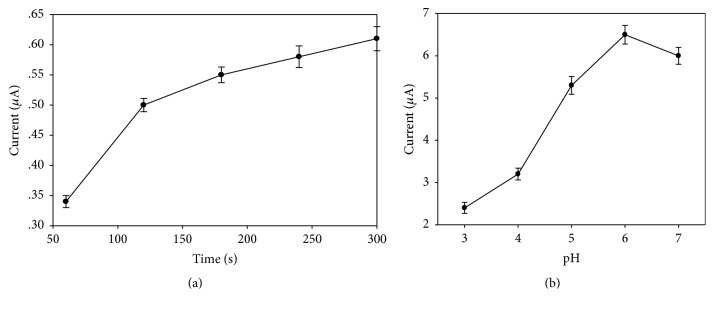
Optimization of test conditions of accumulation time (a) and pH (b).

The dissolution of copper ions on the surface of the electrode mainly is related to preconcentration time and pH value, so these two parameters were mainly optimized. As shown in Figure 5(a), the peak current increased with the enrichment times increasing. But the range of current increase turned smaller after 120 s. If the enrichment time was too long, the electrode surface would be saturated, which might affect the accuracy of the dissolution curve. Therefore, here we select the enrichment time for 120 s. Meanwhile, Figure 5(b) showed when the pH was 6.0, the peak current was strongest, and it was the optimum reaction liquid pH value. So, the optimum test conditions were acetate buffer solution (pH = 6.0) for enrichment of 120 s.

### 4.6. Establishment of Testing Standard Curve

**Figure 6:**
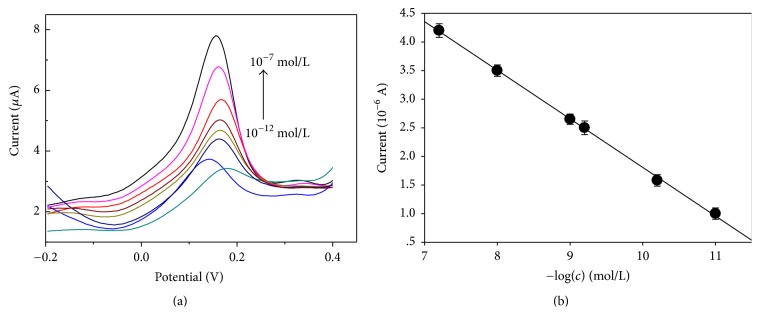
Standard curve of detection of copper based on compound modified electrode. Detection times *n* = 6.

After optimizing the test conditions, we used the optimum system to detect a series of concentrations of copper ions. As shown in Figure 6, the stripping peak current of copper ions increases with the rise of the concentrations of copper ions. The peak current intensity versus the logarithm of copper ion concentration (lg⁡*c*_(Cu2+)_) is linear, and the positive linear correlation is in the concentration range of 10^−11 ^mol/L–10^−7 ^mol/L. The linear equation was *Y* = −0.8496log⁡(*c*) + 10.303, the correlation coefficient was *R*^2^ = 0.9992, and the detect limit of 10^−12 ^mol/L was obtained based on 3*σ* method.

### 4.7. Disturbing Influence of Other Ions

**Figure 7:**
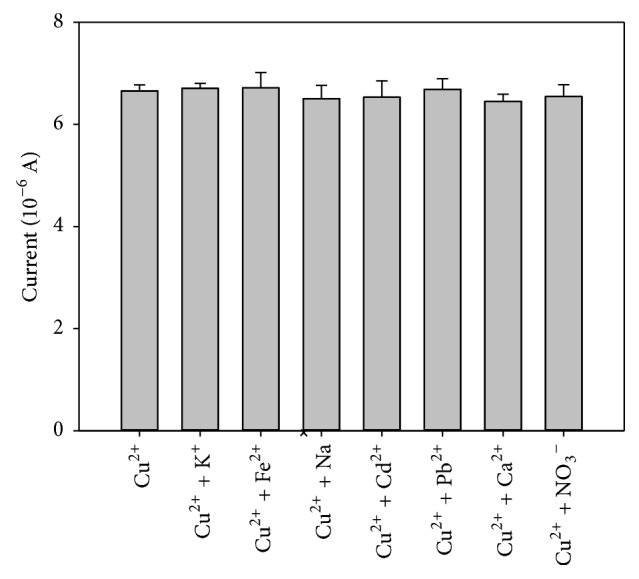
Detection of several ions by compound modified electrode (C_Cu_^2+^ = 10^−8 ^mol/L and other ions are all 10^−7 ^mol/L). Detection times *n* = 6.

The existence of various ions in water may have interference for the detection, so we have detected the disturbing influence of various ions added to copper solution. Under the same detection system, add 10 times the concentration of interfering ions into the solution where copper ion concentration was 10^−8 ^mol/L. The results are shown in Figure 7. The stripping peak has little changed and the signal interference rate is less than 5%. The results show that the composite modified electrodes can achieve the specific detection for copper ion.

### 4.8. Application in River Water Samples

**Figure 8:**
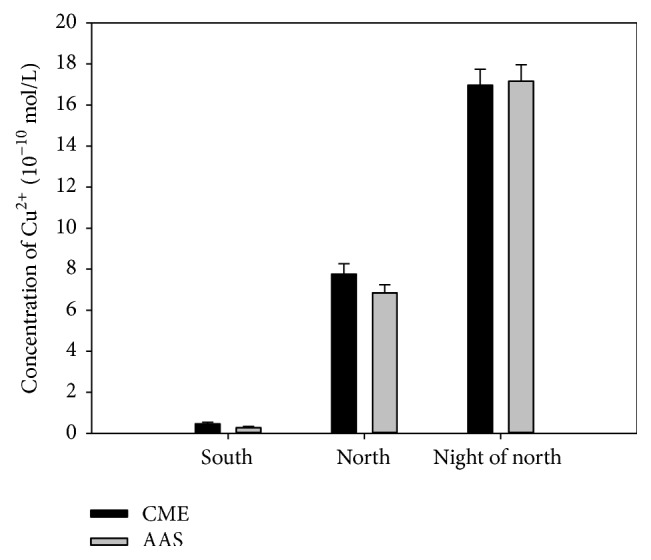
The detection results of actual water samples (CME: compound modified electrode; AAS: atomic absorption spectrometry). Detection times *n* = 6.

Apply this CME to detect the river water samples taken from the Xiangjiang River, respectively, taking the samples from three points of upstream of Changsha section (south), downstream Changsha section (north), and downstream Changsha section at night (night of north). Because the pH of the river water samples was at 5.8–6.2, the same as the pH of detection system, here they do not need to adjust the pH before experiment. As shown in Figure 8, the results detected by CME were consistent with the AAS testing results, showing that the testing results were reliable. From the results, we can find some interest information. The copper ion concentration of downstream of the Xiangjiang River was higher than the upstream and had an obvious increase at night. These data showed that the water quality of the Xiangjiang River water had been polluted after flowing through Changsha city and the pollution was aggravated in the evening. This detection may help us to find the changes of different conditions. Additional, from the testing results, the copper ion concentration in the Xiangjiang River water changes within the 10^−10 ^mol/L level, just in the detection range by this method, and the water does not need pretreatment and is detected directly. All this shows that this method can be used in the daily monitoring of copper ion in river water.

## 5. Conclusions

Modified electrodes based on histidine and gold-labeling multiwalled carbon nanotubes were successfully established. Histidine is specific to the copper ions and gold-labeling multiwalled carbon nanotubes enhance the current signal. This modified electrode can be used for detection of copper ions in river water, and the detection results are consistent with the detection of AAS, but the cost of equipment is cheaper and more convenient to use. In addition, after modification, it can be carried out to do fast, daily monitoring.
